# Block identification and stability analysis of underground stope with multi-working face

**DOI:** 10.1371/journal.pone.0335980

**Published:** 2026-01-06

**Authors:** Minsi Zhang, Chenlong Tie, Bin Wang, Yong Yang, Chen Wang, Wenpan Sun, Jingxiao Xia, Xin Zhou

**Affiliations:** 1 Engineering Research Center for Geological Environment and Underground Space of Jiangxi Province, East China University of Technology, Jiangxi, Nanchang, China; 2 School of Civil and Architecture Engineering, East China University of Technology, Jiangxi, Nanchang, China; 3 The Third Exploration Team of Shandong Coalfield Geologic Bureau, Shandong, Taian, China; 4 Liaoning Institute of Science and Technology, Liaoning, Benxi, China; Henan Polytechnic University, CHINA

## Abstract

The stability analysis of multi-working face stopes presents significant challenges due to complex concave geometries that limit conventional block theory applications. This study proposes a novel methodology in which rock mass models are constructed through the combination of convex sub-regions, enabling the application of traditional cutting algorithms. Block identification is achieved through discontinuity contraction and sub-region merging, effectively accounting for finite discontinuity sizes. The proposed method was implemented into the software GeoSMA-3D and applied to a shallow-buried metal mine stope. The analysis successfully identified all independent blocks, including key blocks critical for stability. The results demonstrate that excavation disturbances lead to an increase in discontinuity persistence, subsequently causing a notable rise in the number of key blocks. The integration of random discontinuities with deterministic features revealed a total of 205 key blocks, 60% of which involved the deterministic discontinuities. These key blocks have a maximum volume of 5.37 m³ and are predominantly distributed along the deterministic discontinuities. The results demonstrate the precision and efficacy of the proposed method for analyzing complex, multi-face excavations. This work provides a robust technical framework for the stability assessment of surrounding rock in mining stopes.

## 1. Introduction

In rock mass engineering, discontinuities hold a crucial role in governing the stability of the rock mass. These discontinuities interact with excavation faces, forming blocks with varying shapes, which significantly impact the overall stability. For excavations featuring simple, regular shapes, traditional block theory effectively simplifies the problem, identifying key blocks and their pertinent information. However, for more intricate underground projects, such as stope with multi-working faces, the interplay between discontinuities and excavation faces becomes intricate, rendering block identification a challenging task. Based on the traditional cutting algorithm, it is very meaningful research to develop new block identification methods that are suitable for complex excavation faces.

The traditional block theory, which conceptualizes natural rock masses as structures comprising numerous discontinuities and rock blocks, offers a distinct perspective from the previous approach that treated rock masses as elastic, homogeneous continua. Block stability analysis methods typically include structural analysis based on stereographic projection, statistical analysis [1,2], and key block analysis [3,4]. The publication of “Block Theory and Its Application to Rock Engineering” by Dr. Shi Genhua and Professor Goodman R.E. in the early 1980s marked the maturation of block theory as an effective analytical tool in rock engineering [5]. According to this theory, hard and semi-hard rock masses are often segmented into spatial blocks with diverse sizes and shapes by discontinuities of varying sequences, types, directions, and properties [6]. These spatial blocks undergo further segmentation due to human engineering excavations, leading to stress redistribution near the excavation face. Under the combined effect of redistributed stress and engineering forces, specific spatial blocks proximal to the excavation face lose their original static equilibrium, becoming key blocks [7]. The instability of key blocks triggers a cascade of failures, ultimately resulting in the collapse of the engineering rock mass. Consequently, analyzing the stability of the engineering rock mass can be reduced to pinpointing and analyzing these key blocks. This suggests that reinforcing solely the identified key blocks is sufficient to maintain the safety and stability of the entire rock mass.

With the unceasing efforts of scholars globally, this method has witnessed increasingly widespread applications, leading to significant advancements in block theory [8,9] For instance, the stability of rock slopes was analyzed using GeoSMA-3D software, designed for spatial block modeling and joint plane simulation [10,11]. A method combining block theory and the Newmark method was introduced to simulate the dynamic stability of rock blocks during earthquakes, aiming to enhance their stability evaluation. Through examples, it demonstrated the effectiveness of this method in simulating the dynamic response and permanent displacement of rock blocks under earthquake conditions [12].

The application of block theory and three-dimensional (3D) block-cutting analysis in underground caverns was compared by Zhang et al. [13]. Subsequently, 3D block-cutting analysis was employed to identify all spatial blocks segmented by finite-sized fractures within the rock mass region. An advanced classic lattice spring model was introduced for probabilistic stability analysis of rock tunnels in low-stress environments, focusing on block fall failures. This model integrated the synthetic rock mass technique and Barton-Bandis joint model, enabling deformable block simulations, tension crack formation, and accurate joint behavior modeling [14]. Budetta discussed the application of both deterministic and probabilistic methods in assessing rock slope stability, comparing and highlighting their respective merits and limitations [15]. A Gaussian Process Regression (GPR) model was established by He P et al. to replace the explicit function of the key block, rapidly predicting the failure probability of the key block by coupling the GPR model with Monte Carlo Simulation (GPR-MCS) [16]. A fast mesh model for the subdivision of tunnel surrounding rock was proposed using a polyhedron-slicing algorithm by Yong Yang et al. Additionally, a new block data structure was designed to simplify slicing calculations [17]. Huidong Wang et al. introduced an extended key block theory that considers inter-block forces and block rotation, integrating it into a 3DEC-based program for more accurate and efficient stability analysis of fractured rock masses [18]. Verification and application examples confirmed the theory’s improved accuracy over traditional methods, emphasizing the importance of considering block rotation and inter-block forces in stability assessments. The block theory was utilized to determine both the removable block type and key block for different excavation surfaces, analyzing the progressive failure of spatial blocks segmented by finite-sized fractures based on their geometric information to gain a deeper understanding of block failure [19]. These studies have significantly contributed to the advancement of block theory. However, research on block identification methodologies tailored specifically for multi-face stopes and accompanying modeling approaches remains limited.

Utilizing the well-established cutting algorithm for convex bodies intersected by planes, this study introduces an innovative approach that combines initial segmentation with subsequent block consolidation, enabling precise block identification. This methodology facilitates the decomposition of complex concave models into multiple convex components, enabling the identification and stability assessment of blocks situated between intricate excavation surfaces and various discontinuities. The proposed method was utilized in a shallow-buried metal mine project, successfully identifying all key blocks on the four working faces at the -40m horizontal section. Based on the calculation results, the study conducts an analysis of the impact of excavation disturbance on the quantity of key blocks and forecasts the dispersal of random blocks by employing random discontinuity parameters. Following this, a stability evaluation for surrounding rock of the mining stope is carried out, thereby offering technical backing for safe mining practices and support design in mines.

## 2. Block identification

The proposed methodology for systematically identifying rock blocks consists of three primary steps: (1) Decomposing the concave volume into convex sub-regions using a face-directed unit method, with the interfaces between these sub-regions defined as auxiliary surfaces for subsequent merging. (2) sequentially introducing discontinuities in descending order of size, with each discontinuity completely cutting all interacting block units. This process inherently enlarges the discontinuities temporarily. (3) Restoring discontinuity sizes and merging blocks separated by auxiliary surfaces or by the temporary over-cutting procedure.

**Fig 1:**
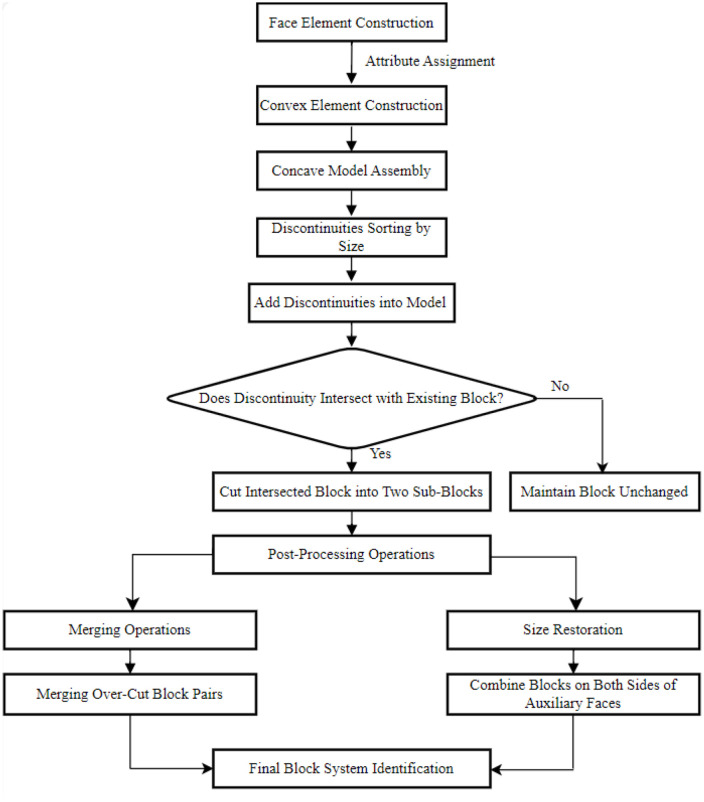
Flowchart of the proposed block identification methodology, outlining the key steps from model construction through discontinuity cutting to block merging and final identification.

The sequence of introducing discontinuities does not affect the final block geometry, as the restoration and merging steps correct any interim over-cutting. However, the order is crucial for computational efficiency. Processing larger discontinuities first allows them to partition the rock mass into major sub-blocks, within which smaller discontinuities are then processed, as illustrated in Fig 1. This strategy minimizes redundant cutting operations that would occur if the sequence were reversed, thereby optimizing the computational workload.

### 2.1. Characterization and morphological analysis for a block

#### 2.1.1. Characterization for a block system.

**Fig 2:**
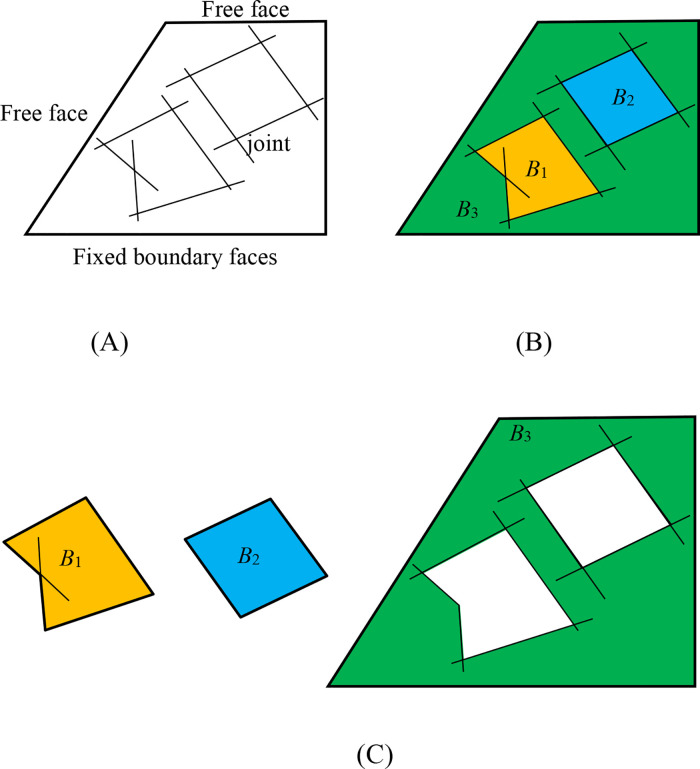
Schematic diagrams illustrating the formation and classification of rock blocks, including finite/infinite types and their unified polyhedral representation. **(A)** Rock mass model with discontinuities cutting to form potential blocks; **(B)** Block system showing classification into finite (B1, B2) and infinite blocks (B3); **(C)** System elements demonstrating unified polyhedral representation with directed faces and edges.

Rock blocks are formed by the intersection of discontinuities, with their boundaries defined by discontinuities, free faces (excavation surfaces), or fixed boundary faces of the model, as illustrated in Fig 2(A). Block theory categorizes blocks into two types: finite blocks and infinite blocks. Finite blocks, isolated in space, are completely detached by discontinuities and free faces. These may exhibit either concave polyhedral geometry (e.g., Block B_1_ in Fig 2(B)) or convex polyhedral geometry (e.g., Block B_2_ in Fig 2(B)). From a kinematic perspective, finite blocks are further classified into immovable blocks and movable blocks. Infinite blocks, in contrast, remain partially connected to the rock mass and are not fully separated by discontinuities or free faces. In numerical modeling, such blocks are characterized by the inclusion of fixed boundary faces, as exemplified by Block B_3_ in Fig 2(B).

In this study, the geometric modeling of blocks does not differentiate between these two categories. As shown in Fig 2(C), all blocks (*B*_1_, *B*_2_, and *B*_3_) are uniformly represented as polyhedrons. Within the block system, blocks serve as fundamental elements, and the properties of discontinuities are manifested by the face of the blocks. A block is composed of directed faces, each consisting of directed edges with specific directional definitions (as detailed in Section 2.1.2).

(1) Characterization of discontinuities. Discontinuities can be categorized based on their spatial extension range. The first category includes discontinuities with a limited extension range, encompassing both deterministic and stochastic discontinuities. These planes are conceptualized as planar disks and are described by parameters including dip angle, dip direction, diameter, and the precise three-dimensional coordinates of the disk’s center. The second category comprises discontinuities that can be considered as extending infinitely within the study scope, such as faults. These planes are characterized by their dip angle, dip direction, and the three-dimensional coordinates of any point on the plane.

Rock masses are bounded by discontinuities, making the characterization of discontinuities essential for geological analysis. However, in practical applications, the concealed nature of discontinuities within rock masses poses significant challenges in accurately measuring their geometric configurations. To address this limitation, scholars have proposed various idealized assumptions, including the Dershowitz polygonal model, elliptical disc model, and Baecher disc model. Among these, the Baecher disc model has gained widespread acceptance by conceptualizing discontinuities as non-thickness planar circular discs. This study adopts the Baecher disc model, which can be mathematically represented by the following formulation:


$$\left\{ \;\;\;\begin{array}{c} A\cdot XT+d=0\\ {\left(x-x_{c}\right)}^{2}+{\left(y-y_{c}\right)}^{2}+{\left(z-z_{c}\right)}^{2}\leq r^{2}\\ A=(sin\alpha \cdot \cos(\pi /2-\beta ),-\sin\alpha \cdot \sin(\pi /2-\beta ),cos\alpha ) \end{array}\right.$$ (1)

where *α* and *β* denote the dip direction and dip angle of a discontinuity, respectively, *d* represents the planar positioning parameter of the disc, *r* is the disc radius, and (*x*_c_, *y*_c_, *z*_c_) corresponds to the coordinates of the disc center. Notably, when the radius *r* is assigned a value significantly exceeding the dimensions of the rock mass model, the discontinuity is regarded as a persistent discontinuity plane (i.e., fully traversing the model domain).

(2) Characterization of a block. In the present study, a consistent data storage format is employed for both complex rock mass models and blocks bounded by discontinuities. They can be represented as a set of directed polygons, each consisting of multiple directed edges that are connected end-to-end.
(3) Characterization of faces within a block. In this study, block faces are categorized into four types based on their distinct functions: free faces, fixed faces, virtual faces, and cutting faces. Within the initialized rock mass model, free faces and fixed faces are inherently present. Free faces, defined as faces in direct contact with open space, serve as a necessary condition for block movability. Conversely, fixed faces constitute all other boundaries excluding the free faces, bridging the model with the rock mass beyond the study scope. Virtual faces emerge during the segmentation of concave models into convex shapes; these faces are not physically existent and vanish once the cutting process concludes, facilitating the merging of blocks. Cutting faces, on the other hand, arise from the intersection of discontinuities with blocks, collaboratively forming the boundaries of individual blocks alongside the free faces.

#### 2.1.2. Morphological analysis for a polygon.

**Fig 3:**
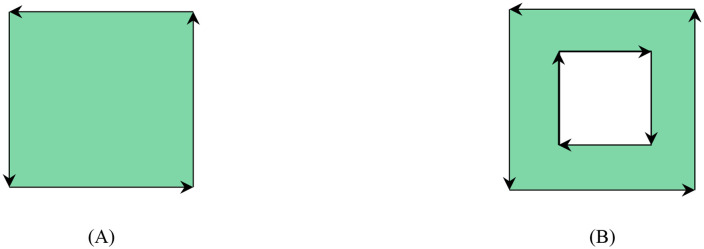
Convention for defining edge directions in polygons, which is fundamental for geometric calculations. **(A)** Direction of edges in a convex polygon **(B)** Direction of edges in a concave polygon.

Taking planar polygons as an example for analysis, this methodology can be extended to polygons in three-dimensional space. In a two-dimensional polygon, edge orientations are topologically defined by their sequential connectivity, establishing consistent chirality (clockwise or counterclockwise) through the vertex ordering sequence. The orientation of each edge extends from its starting point to the starting point of the subsequent edge, while the terminal edge’s orientation connects its starting point to the initial edge’s starting point. For polygons containing interior cavities, the orientation of the inner boundaries (cavity edges) is opposite to that of the outer boundary. As clearly illustrated in Fig 3, this schematic diagram demonstrates the directional relationship between the edges of the polygons, where the exterior contour follows a counterclockwise orientation while interior cavities adopt clockwise orientations.

(1) The directional relationship between edges and face

The face orientation is represented by its normal vector, with the positive direction pointing toward the interior of the block. By sequentially numbering edges starting from an initial edge (labeled 1) to a terminal edge (labeled *m*), the relationship between edge orientations and face orientation in a convex polygon satisfies the following equation:


$$\left(n_{i}\times n_{i+1}\right)\cdot n_{p}>0$$ (2)

Where $n_{i}$ denotes the unit direction vector of an arbitrary edge, $n_{i+1}$ represents the unit direction vector of the subsequent edge, and $n_{p}$ is the unit normal vector of the face.

(2) Concave-Convex region discrimination

In concave polygons, adjacent edges at convex angles satisfy Equation (2), while those at concave angles exhibit an inverse relationship. Thus, Equation (3) also serves as a criterion for distinguishing convex and concave angles. Based on this framework, edges in a closed loop can be sequentially ordered. Once a single convex angle is identified, the orientations of all edges can be determined, enabling systematic classification of angular concavity and convexity. In a planar polygon, if the vertex coordinate *X*_*i*_ satisfies Equation (3), the corresponding angle at this vertex can be identified as convex.


$$X\in \max\left(\left\{X_{i}|i=1,2,3,...,m\right\}\right)\cup \min\left(\left\{X_{i}|i=1,2,3,...,m\right\}\right)$$ (3)

where *X*_i_ represents either the *X*-coordinate or *Y*-coordinate of the vertex under evaluation.

(3) Area calculation for a polygons

In a plane, select an arbitrary point as the base vertex. By connecting this base vertex to all edges of the polygon, a series of triangles is formed. The algebraic sum of the areas of these triangles yields the area of the polygon, where triangle areas may be assigned negative values. For computational convenience, the origin *O* is typically chosen as the base vertex. Assuming the polygon has edges sequentially defined by vertices *V*_1_, *V*_2_, …, *V*_*n*_, its area can be calculated using the following formula:


$$S_{P}=\sum_{i=1}^{n}\frac{\left({{OV}_{i}\times n}_{i}\right)\cdot n_{P}}{\left|\left({OV}_{i}\times n_{i}\right)\cdot n_{P}\right|}\cdot S_{i}$$ (4)

where, $S_{i}$ represents the signed area of the triangle formed by the *i*-th edge and point *O*; $n_{i}$ denotes the direction vector of the *i*-th edge; and $n_{P}$ corresponds to the orientation vector of the polygon.

#### 2.1.3. Morphological analysis for a polyhedron.

Similar to the properties of polygons, a polyhedron can be analyzed using analogous methods for determining convexity/concavity and computing their volumes.

(1) Orientation relationship between adjacent faces

**Fig 4:**
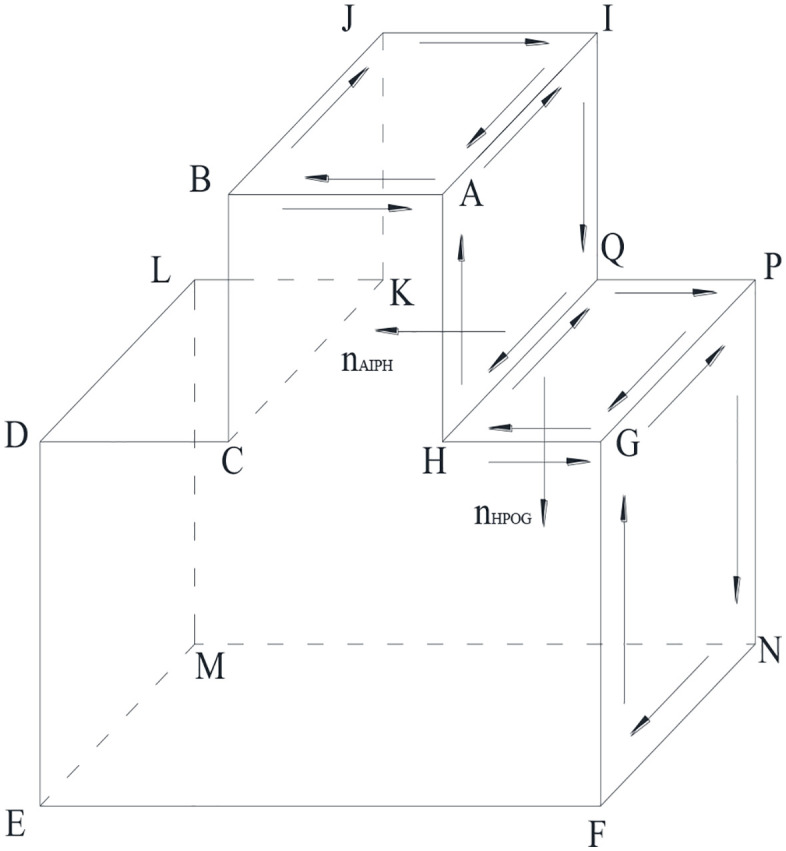
The topological rule showing the reversed direction of a shared edge between two adjacent faces in a polyhedron.

In a polyhedron, a single edge is shared by two intersecting faces, and the direction of this edge is reversed between the two faces (as shown in Fig 4). Consequently, once the orientation of any face within the polyhedron is determined, the orientations of all other faces and edges can be derived.

(2) Determination of convex and concave regions

For a polyhedron, a vertex is classified as convex if it satisfies the following condition:


$$X\in \max\left(\left\{X_{i}|i=1,2,3,...,m\right\}\right)\cup \min\left(\left\{X_{i}|i=1,2,3,...,m\right\}\right)$$ (5)

Where *X*_*i*_ denotes the coordinates of the *i*-th vertex of the polyhedron. The analyzed region is formed by the intersection of two adjacent faces. Let $n_{P1}$ and $n_{P2}$ represent the orientation vectors of the two intersecting faces, and $n_{1}$ denote the directional vector of the shared edge within the first face. The region is identified as concave if the following condition holds:


$$\left(n_{P1}\times n_{P2}\right)\cdot n_{1}>0$$ (6)

Otherwise, the region is classified as convex.

(3) Block volume calculation

By selecting the coordinate origin *O* as a common vertex, pyramids are formed by connecting *O* to each face of the polyhedron. The volume of a polyhedron can be calculated as the algebraic sum of the volumes of pyramids formed by connecting the coordinate origin (as the common vertex) to each face of the polyhedron, where the volumetric contributions of individual pyramids may include negative values. For faces with orientation vectors $n_{P1}$, $n_{P2}$, …, $n_{Pn}$, let *V*_*i*_ denote the volume of the pyramid formed by face *i* and vertex *O*. The total volume *V* of the polyhedron is then given by:


$$V=\sum_{i=1}^{n}\frac{A{}_{i}O\cdot n_{Pi}}{\left|A_{i}O\cdot n_{Pi}\right|}\cdot V_{i}$$ (7)

### 2.2. Constructing a rock mass model

Modeling stands as a cornerstone for all numerical analysis methods, with different techniques requiring tailored modeling approaches. This process involves thoroughly characterizing all elements being studied. Specifically, in the realm of block theory analysis, these elements include initial rock mass models, discontinuities, and the blocks resulting from their intersection. In this study, a methodology is adopted that involves constructing a convex body model using directed face elements. Furthermore, a detailed framework for creating complex concave polyhedra is devised by amalgamating several convex bodies. The rock mass model is essentially an initialization of the block system, wherein all blocks formed after cutting calculations stem from the rock mass model and inherit its inherent properties.

(1) Convex model construction

In this study, the rock mass model is constructed employing the directed face element method, which fundamentally represents the rock mass as a polyhedron. Each face of the polyhedron is composed of edges connected sequentially end-to-end. The relationship between the direction of the edges and the orientation of the face follows the right-hand rule. The face orientation is defined by its normal vector, which is designated as positive when pointing toward the interior of the block. Faces within the rock mass model are systematically classified into three distinct categories based on their geological and functional characteristics: free faces, fixed faces, and auxiliary faces. Each category is defined as follows:

A free face is defined as an exposed face that is in direct contact with the atmosphere. It serves as a necessary condition for the formation of key blocks, as it provides a boundary where rock displacements may occur. A fixed face represents the predefined boundary of the study domain. In practice, such faces are connected to the surrounding rock mass, constraining the model and simulating continuity with the larger geological formation. An auxiliary face is a virtual interface introduced during the construction of concave models via the assembly of convex sub-regions. It constitutes a common surface between adjacent sub-blocks and is used to partition concave geometries into simpler convex units. It is critical to note that auxiliary faces are computational artifacts and are not physically present in the actual engineering structure. This classification supports the systematic analysis of block stability and facilitates the accurate representation of complex geological geometries in numerical simulations.

**Fig 5:**
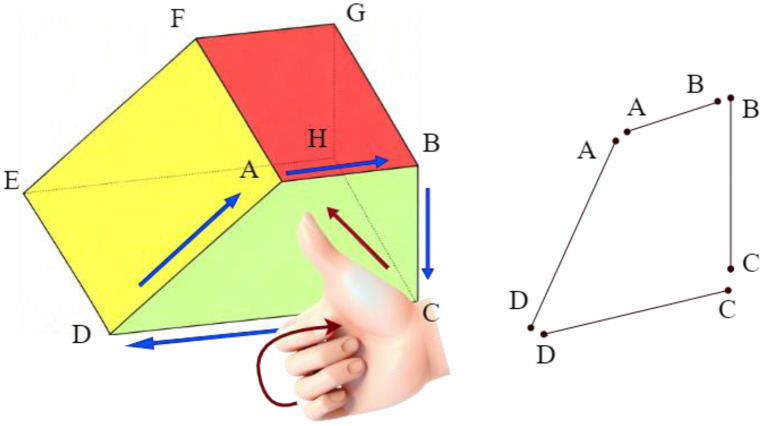
Edge-face direction relationship in a convex polyhedral model, illustrating how faces are composed of sequentially connected edges with orientations determined by the right-hand rule.

As illustrated in Fig 5, the polyhedral block comprises six faces. For instance, face ABCD is formed by edges AB, BC, CD, and DA connected in sequence. During data storage, the spatial coordinates of the vertices and their sequential order determine the direction of each edge. Subsequently, the face orientation is derived by applying the right-hand rule to the ordered edges. The detailed computational methodology for determining these orientations is described in Section 2.1.3. Table 1 shows the composition of all the faces.

**Table 1:** Building of convex polyhedron model.

Face			Vertex order
Number	Name	Properties	
1	ABCD	Fixed face	A→B→C→D
2	GFEH	Fixed face	G→F→E→H
3	EDCH	Fixed face	E→D→C→H
4	EFAD	Free face	E→F→A→D
5	BGHC	Fixed face	B→G→H→C
6	FGBA	Free face	F→G→B→A

(2) Concave model construction

Conventional planar cutting algorithms are exclusively applicable to convex polyhedra and cannot be effectively extended to concave polyhedra. Moreover, the direct application of planar cutting techniques to concave polyhedra proves computationally intensive and exhibits poor robustness. To overcome these limitations, this study employs a decomposition strategy where auxiliary planes are strategically introduced to dissect concave blocks into a set of simpler convex sub-regions. These auxiliary planes act as virtual boundaries that segment the original concave volume into computationally manageable convex units, enabling the application of robust convex-based algorithms.

During model construction, these convex sub-blocks are assembled to reconstruct the original concave geometry. The virtual faces between adjacent convex units are eliminated in the final merging process, resulting in a seamless representation of the complex shape. This method effectively circumvents the direct cutting of concave bodies while maintaining mathematical rigor throughout the identification process. The implementation follows a systematic procedure where models are first created in CAD software, with different faces labeled using distinct layers, and then exported in DXF format for programmatic processing. The integration of auxiliary planes thus provides a practical framework for handling realistic geological features without compromising computational stability, forming the foundation for the advanced block identification methodologies discussed in subsequent chapters.

**Fig 6:**
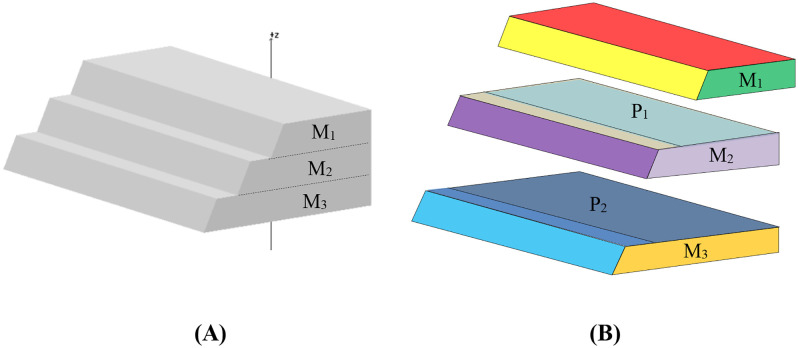
Construction of a concave stope model by combining three convex sub-regions (M1, M2, M3). **(A)** Final merged excavation geometry after eliminating virtual boundaries. **(B)** Display of sub-blocks of the combined slope.

For concave configurations, Fig 6 demonstrates a multi-step slope model constructed through the combination of three convex bodies (M₁, M₂, M₃).The interfaces between these bodies, including the bottom face of M₁ (P₁ within M₂) and face P₂ within M₃, are initialized as virtual faces. These temporary surfaces ensure proper geometrical representation during the cutting phase but are eliminated during the final merging process (detailed in Section 2.3), resulting in a unified model that accurately reflects the actual excavation geometry. This approach effectively circumvents the direct cutting of concave bodies while maintaining mathematical rigor throughout the identification process.

### 2.3. Block merging algorithm

Blocks consist of polygons, decomposable into directed edges. Block merging is the consolidation of directed faces, reducible to directed edge merging. Thus, a block merging approach is proposed based on directed edge merging for concise and efficient operations. When two blocks contact each other in a face-to-face manner, the merging process follows the methodology described in this section. The essence of merging is the union of two coplanar faces with overlapping regions. For faces with the same orientation, merging requires coincident edges;for faces with opposing orientations, merging requires overlapping face areas. Non-coplanar blocks cannot be merged.

(1) Merging Rules

Rule R1: Delete two overlapping faces with opposing orientations

Rule R2: Delete two overlapping edges with opposing orientations.

Rule R3: Merge collinear edges connected end-to-end sequentially. The merged edge extends from the start point of the first edge to the end point of the second edge.

(2) Merging Procedure

The face merging process requires initial topological harmonization between Face A and Face B. This is achieved through a bidirectional subdivision procedure: each edge of Face A that contacts Face B is extended to subdivide Face B (or its sub-faces), and conversely, each contacting edge of Face B is used to subdivide Face A. This mutual cutting ensures geometric compatibility prior to the application of the merging rules R1–R3. The orientations of sub-faces and their edges remain unchanged. Combine all sub-faces and apply Rule R1. Group the remaining sub-faces and apply Rules R2 and R3 to coplanar edges. The final result is a composite face formed by sequentially connected edges.

(3) Case Demonstrations

**Fig 7:**
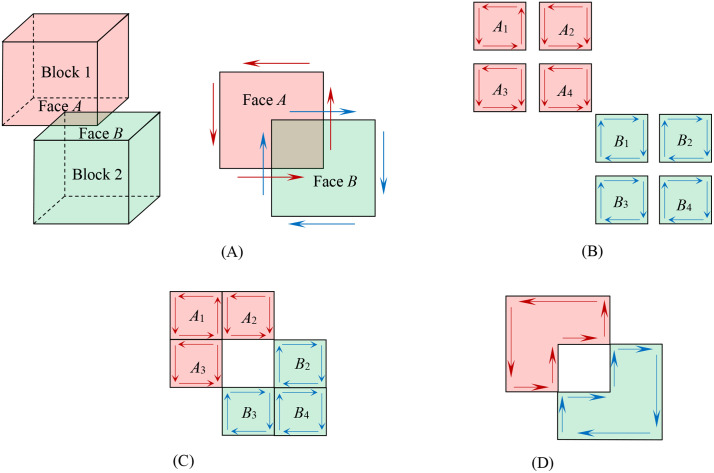
Sequence showing the merging of two overlapping faces with opposite orientations based on directed edge operations. **(A)** Initial State: Two adjacent blocks with overlapping faces that have opposing orientation vectors. **(B)** Subdivision: Each face is subdivided by projecting the edges of the opposing face, creating multiple sub-faces. **(C)** Deletion: The overlapping sub-faces with opposite orientations are deleted according to Rule R1. **(D)** Final Merged Face: The remaining sub-faces are merged using Rules R2 and R3 to form a single composite face, while non-overlapping regions remain unchanged.

Merging of Oppositely Oriented Faces. As illustrated in Fig 7(A), faces A (on Block 1) and B (on Block 2) overlap. First, all edges of Face A were traversed to subdivide Face B into four distinct subfaces (B1–B4) as shown in Fig 7(B). Reciprocally, the same edge traversal method was applied to Face A, generating four subfaces (A1–A4). Rule R1 was then implemented to eliminate overlapping regions between subfaces A4 and B1 as shown in Fig 7(C). Subsequent application of Rules R2 and R3 to the remaining subfaces (A1–A3 and B2–B4) produced the unified merged structure shown in Fig 7(D). Notably, non-overlapping regions of both blocks remained unmodified throughout the process.

**Fig 8:**
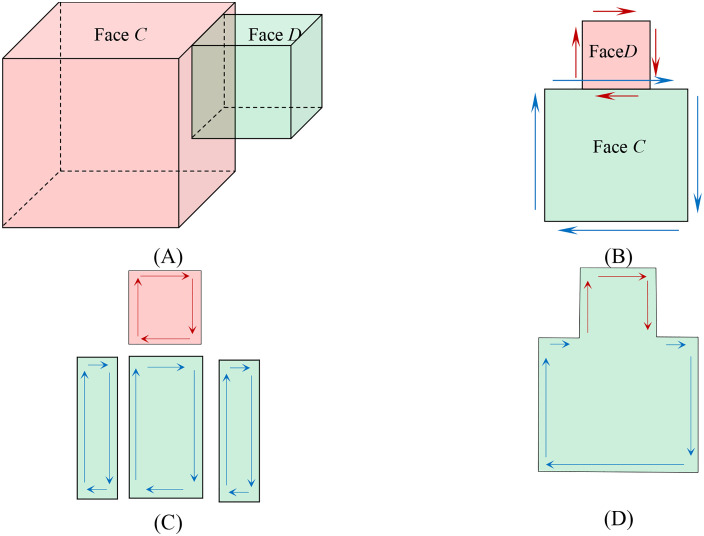
Sequence illustrating the merging of two overlapping faces with the same orientations based on directed edge operations. **(A)** Initial state with two adjacent blocks. **(B)** Illustration of edge directions within a face. **(C)** Subdivision of faces by edges **(D)** Final merged face forming a concave polyhedron.

Merging of Same Oriented Faces. As illustrated in Fig 8(A), Faces C (on Block 1) and D (on Block 2) are coplanar and share coincident edges along their interface. Initially, the edges of Face C were traversed; however, due to the geometric alignment, no subdivision of Face D occurred as shown in Fig 8(B). Conversely, traversing the edges of Face D resulted in the subdivision of Face C into three distinct subfaces (C1–C3), as depicted in Fig 8(C). Rule R3 eliminated redundant edges generated during the partitioning. The subsequent integration of all valid edges yielded the merged configuration shown in Fig 8(D). Throughout the process, non-interacting regions of both blocks remained unaltered, thereby completing the merging of the two blocks while preserving their external geometric integrity.

### 2.4. Block identification method

Currently, there are two primary methods for 3D block identification: intersection loop analysis and polyhedron combination algorithm. This paper presents an improvement to the latter approach, recognizing blocks between arbitrary discontinuities through a process of cutting and subsequent merging. Distinct algorithms have been devised specifically for convex and concave models. Additionally, a novel block merging method is introduced based on directed edge merging, which integrates the merging of faces with the same and different directions into one unified algorithm.

#### 2.4.1. Block identification method in convex model.

Initially, all discontinuities within the study area are uniformly numbered. Then, these discontinuities are sequentially integrated into the existing block system based on their numbering order. When incorporating the first discontinuity, the existing block system represents the pre-established rock mass model.

**Fig 9:**
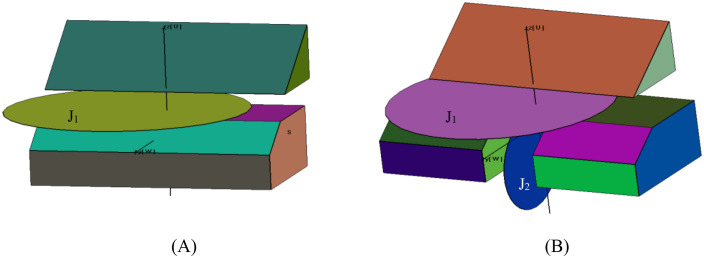
Sequential cutting of a rock mass model by discontinuities, demonstrating the block subdivision process. **(A)** Initial intersection of discontinuity J1, dividing the rock mass into two distinct sections (A1 and A2). **(B)** Subsequent introduction of discontinuity J2, which further partitions the lower block while leaving the upper block unaffected.

Secondly, whenever a new discontinuity is introduced, all existing block bodies need to be evaluated. The discontinuity cuts the intersecting block into two sub-blocks. As illustrated in Fig 9, there are two joints J_1_ and J_2_ in slope engineering. When J_1_ is introduced into the model, it intersects with the rock mass model, dividing it into two sections, as shown in Fig 9(A). With the addition of J_2_, it intersects only with the block below J_1_, further dividing it into two parts. Since J_2_ does not intersect with the block located above J_1_, that block remains unaffected, as illustrated in Fig 9(B).

Finally, based on the assumptions of the cutting algorithm, the size of the discontinuity is temporarily enlarged. Once the cutting process for all discontinuities within the block system is completed, the discontinuity is restored to its original size. This restoration leads to the merging of some blocks, facilitating the identification and search of the actual blocks.

**Fig 10:**
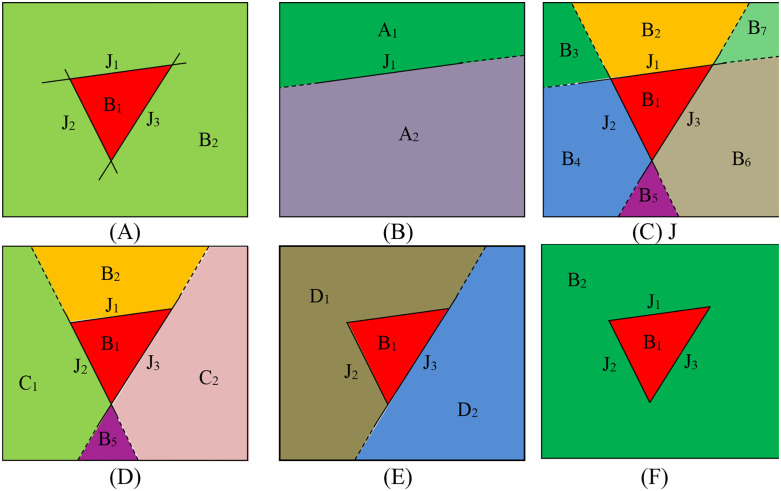
Sequential block identification process in a convex polyhedron model using a 2D analogy. **(A)** Initial rock mass with discontinuities J_1_-J_3_, **(B)** Division after J_1_ cut, **(C)** Further division with J_2_ and J_3_ cuts, **(D)** Merge after J_1_ size restoration, **(E)** Additional merge after J_2_ restoration, **(F)** Final block configuration after J_3_ restoration.

In order to express the process of block identification clearly, a two-dimensional diagram is used instead of a three-dimensional one. As shown in Fig 10(A), the rock mass model is represented as a rectangle intersected by three discontinuities, labeled J_1_ to J_3_, which divide the rock mass into two parts, B_1_ and B_2_. Firstly, the plane-cutting-polyhedron algorithm is employed to sequentially introduce three discontinuities into the model. According to the assumption of the cutting algorithm, the discontinuity completely severs the block intersecting with it. After adding J_1_, the rock mass model is bisected into two sections, A_1_ and A_2_, as depicted in Fig 10(B). The sequential introduction of J_2_ and J_3_ further divides the rock mass into seven sections, labeled B_1_ to B_7_, as shown in Fig 10(C). Obviously, the calculated dimensions of the discontinuity are enlarged to the size indicated by dashed lines, whereas the actual dimensions are denoted by solid lines.

Once the cutting process is complete, the three discontinuities are reduced to their actual size. After shrinking J_1_, an overlapping area emerges between B_3_ and B_4_, indicating incomplete separation. This suggests that, in practical engineering scenarios, these two sections belong to the same block; thus, they are merged into C_1_, while B_6_ and B_7_ are combined into C_2_, as illustrated in Fig 10(D). Continuing to shrink J_2_ leads to the merging of B_2_ and C_1_ into D_1_, and B_5_ and C_2_ into D_2_, as shown in Fig 10(E). Finally, shrinking J_3_ results in the amalgamation of D_1_ and D_2_ into B_2_, ultimately achieving block identification. The outcome presented in Fig 10(F) corresponds to the real situation shown in Fig 10(A).

#### 2.4.2. Block identification method in concave model.

The robustness of the program may be affected due to the wide variety of polyhedra and the complexity of cutting situations. In this study, the complex concave model is divided into several simple convex bodies. The structure plane cuts the convex bodies after the segmentation and then recombines them after the cutting is completed.

**Fig 11:**
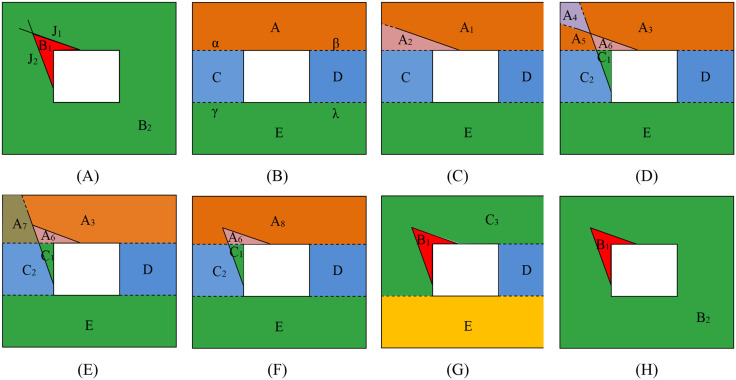
Block identification process in a concave polyhedron model through convex decomposition. **(A)** Initial concave excavation geometry, **(B)** Division into convex sub-regions (A,C,D,E) with auxiliary faces (α,β,γ,λ), **(C)** Cutting by discontinuity J_1_, **(D)** Further division by J_2_, **(E)** Block merging after J_1_ shrinkage, **(F)** Additional merging after J_2_ shrinkage, **(G)** Elimination of auxiliary face α, **(H)** Final block configuration after removing all auxiliary faces.

As shown in Fig 11(A), the study area is a rectangle with a rectangular excavation face. There are two non-penetrating discontinuities J_1_ and J_2_ in the model, which form a block B_1_ with the excavation face. The block identification method is briefly described as follows:

(1) The rock mass model is partitioned into four distinct convex regions labeled A, C, D, and E. This partitioning results in the formation of four new faces, namely α, β, γ, and λ, as depicted in Fig 11(B).
(2) The introduction of discontinuity J_1_ serves to segment the existing block system. Specifically, J_1_ interacts solely with region A, dividing it into two sub-blocks, A_1_ and A_2_, while leaving the remaining blocks unaffected. This configuration is illustrated in Fig 11(C).
(3) The subsequent addition of discontinuity J_2_ further partitions the blocks. Specifically, J_2_ dissects A_1_ into A_3_ and A_4_, A_2_ into A_5_ and A_6_, and C into C_1_ and C_2_, as visualized in Fig 11(D).
(4) Upon restoring J_1_ to its original size, sub-blocks A_4_ and A_5_ coalesce to form a single block, labeled A_7_, as shown in Fig 11(E). Similarly, when J_2_ is retracted to its original position, A_3_ and A_7_ combine to form A_8_, as depicted in Fig 11(F).
(5) Auxiliary faces α, β, γ, and λ are deleted, resulting the existing blocks to merge. Upon the removal of face α, C_2_ and A_8_ merge into C_3_, while C_1_ and A_6_ combine to constitute the identified cone-shaped block, labeled B_1_, as seen in Fig 11(G). The subsequent elimination of faces β, γ, and λ results in the consolidation of C_3_, D, and E into a single block, labeled B_2_, as illustrated in Fig 11(G). This approach circumvents the direct cutting of concave bodies by discontinuities.

### 2.5. Stability analysis

A block is deemed unstable if it satisfies three criteria: the presence of an exposed surface, geometric movability, and a stability coefficient that falls below a specified threshold. The stability coefficient is determined using the Mohr-Coulomb failure criterion, with the active force limited to the block’s self-weight. This assessment can be divided into two distinct cases:

(1) When a block slides along a single face, labeled *i*, the stability coefficient is calculated as follows:

$$k=\frac{Q\cos\alpha_{i}\cdot \tan\varphi_{i}+C_{i}S_{i}}{Q\sin\alpha_{i}}$$ (8)

Where *Q* is the weight of the block, *α*_*i*_ is the inclination angle of the sliding face, *S*_*i*_ is the area of the sliding face *i*, *C*_*i*_ and *φ*_*i*_ are the cohesion and internal friction angle of the sliding face respectively.

(2) When a block slides along two intersecting faces, labeled *i* and *j*, the stability coefficient is computed using the following formula [16]:

$$k=\frac{N_{i}\tan\varphi i+N_{j}\tan\varphi j+C_{i}S_{i}+C_{j}S_{j}}{Q\sin\alpha }$$ (9)

Where *N*_*i*_ and *N*_*j*_ are the normal stresses acting on the two sliding faces; *C*_*i*_, *φ*_*i*_ and *C*_*j*_, *φ*_*j*_ are the cohesion and internal friction angle on the sliding face *i* and *j* respectively; *S*_*i*_ and *S*_*j*_ are respectively the areas of the sliding faces *i* and *j*; Q is the weight of the block; *α* is the inclination angle of the intersection line between the sliding faces *i* and *j*.

### 2.6. Visual program development

To implement the proposed algorithm and facilitate its engineering applications, this study developed a software framework in the VC++ environment and integrated the OpenGL (Open Graphics Library) library to enable 3D visualization. Through the synergistic application of these technologies, a visual block analysis software named GeoSMA-3D was successfully developed. This software not only enhances the practicality of the algorithm but also improves user interaction, providing a powerful tool for engineering analysis.

## 3. Engineering applications

### 3.1. Engineering background

A shallow-buried metal mine located in Zunhua City, Hebei Province, China, serves as the focus of this study. The mining area is characterized by the widespread distribution of metamorphic rock series belonging to the Archaean Qianxi Group. The geological structure of the mining area exhibits a monoclinal configuration, with the overall stratum oriented nearly north-south and tilting westward at an inclination angle ranging from 60° to 80°. The rock mass, primarily composed of biotite amphibole plagioclase gneiss, has a density of 2.65 g/cm³, as determined by laboratory tests. The in-situ stress field is dominated by gravitational forces, given the shallow burial depth of the stopes. Although the -40m level is relatively dry, the potential weakening effect of groundwater on joint shear strength was considered in a conservative approach during stability calculation

The mine being studied is currently operational and actively engaged in production. Recently, there has been a significant escalation in mining intensity and scale, resulting in progressively intricate mining faces and the subsequent need for pillar recovery in later mining phases. These conditions render the stope prone to various instabilities, including roof falling, caving, and collapse, posing significant challenges to mine production safety. Furthermore, the joint development observed in the -40m horizontal middle section of the mining area has a pronounced impact on the ore body, particularly in the northern region where the ore body thickness is more substantial. Therefore, assessing the stability of the rocks surrounding the stope and adopting vital support measures becomes paramount to prevent accidents and ensure the safe mining of ore pillars.

### 3.2. Analysis results

#### 3.2.1. Basic information.

**Fig 12:**
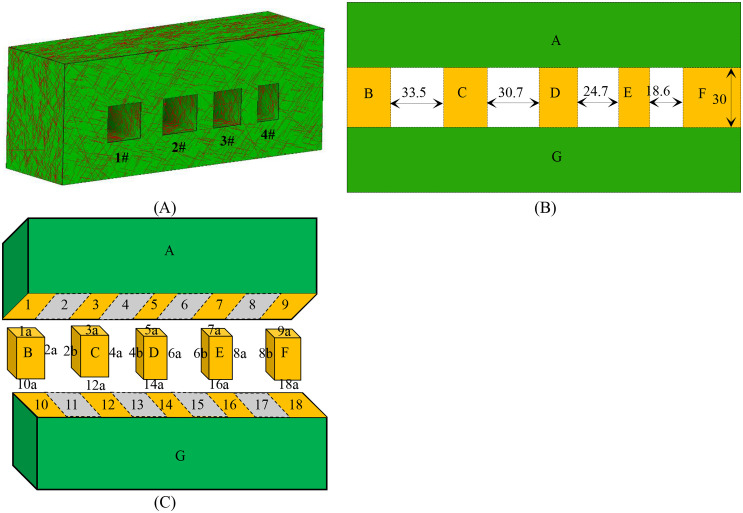
Three-dimensional model of the stope at the -40m horizontal level, showing the excavation geometry with convex polyhedra. **(A)** -40m horizontal middle goaf model **(B)** Goaf model segmented into 7 convex parts (A-G), illustrating the initial rock mass division. **(C)** Three-dimensional view showing the convex decomposition (parts A-G) and attributes of the sub-regional face units.

This study focuses on four stopes located in the midsection of the -40m horizontal mining area, designated as 1#, 2#, 3#, and 4#. These stopes exhibit spans of 33.5m, 30.7m, 24.7m, and 18.6m, respectively, with a uniform height of 30m and depth of 20m. Fig 12(A) illustrates the goaf model, constructed using convex polyhedral. Fig 12(B) presents a front view of the model decomposition and associated dimensions. Furthermore, Fig 12(C) illustrates the decomposition pattern in a three-dimensional diagram, along with the attributes of the sub-regional face units. Detailed face unit attributes are provided in Table 2.

**Table 2:** Decomposition of the model.

Face Type	Subsegment						
	A	B	C	D	E	F	G
Free face	2,4,6,8	2a	2b,4a	4b,6a	6b,8a	8b	11,13,15,17
Auxiliary face	1,3,5,7,9	1a,10a	3a,12a	5a,14a	7a,16a	9a,18a	10,12,14,16,18
Fixed face	Remainder	Remainder	Remainder	Remainder	Remainder	Remainder	Remainder

The actual rock mass represents a three-dimensional configuration obtained through the vertical projection of this figure into the interior. The mine employs an open stope mining method, with 1# and 4# stopes excavated in the first phase and 2# and 3# stopes in the second phase. Following the completion of the first mining phase, field surveys revealed the presence of a few large-scale deterministic discontinuities alongside numerous small-scale discontinuities that are challenging to accurately enumerate. Table 3 summarizes the development of the deterministic discontinuities. The average increase in trace length of 3.2 m after the second mining phase was derived from a comparative analysis of detailed scanline surveys conducted in the same access drift before Phase 1 and after Phase 2 excavation. This quantitative measure of excavation damage provides a critical input for modeling the evolution of block stability.

**Table 3:** The features of deterministic discontinuities.

Group number	Attitude/°	Length/m	Shear strength	
			*φ*/°	C/MPa
**1**	165 ~ 194∠48 ~ 63	18 ~ 25	36	0.16
**2**	281 ~ 312∠66 ~ 82	15 ~ 20	38	0.15
**3**	46 ~ 62∠52 ~ 61	19 ~ 26	41	0.18

**Fig 13:**
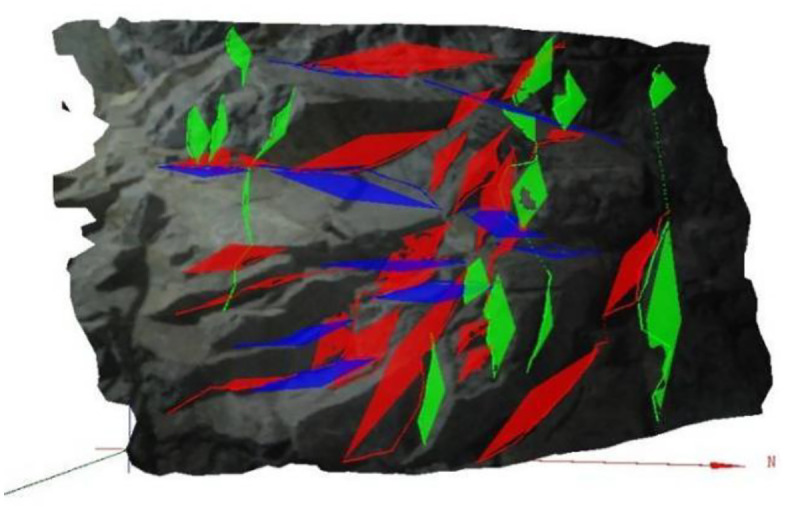
Field measurement results of discontinuities using photogrammetry, showing the spatial distribution of distinct groups.

Utilizing photogrammetry technology, the parameters of numerous random discontinuities, characterized by their small size, were captured and statistically analyzed according to established principles. Fig 13 depicts the measurement results, wherein various colors represent distinct groups of discontinuities. A comprehensive statistical analysis was conducted on the information of each group, yielding crucial insights into the dip direction, dip angle, trace length, spacing, and linear density of the discontinuities. The analysis revealed that the attitude (comprising dip direction and dip angle) of the discontinuities adheres to normal and uniform distributions, whereas the trace length and spacing conform to a negative exponential distribution. The simulation parameters derived from this analysis for the random discontinuities are presented in Table 4.

**Table 4:** The parameters of discontinuities in network simulation.

Group number	Dip angle		Dip direction		Trace length	Spacing
	Mean value/°	Standard deviation	Mean value/°	Standard deviation	Mean value/m	Mean value/m
**1**	65.5	13.7	229	8.9	1.5	0.9
**2**	37.5	12.8	67.9	11.6	1.8	1.7
**3**	84.6	4.3	96.2	9.3	1.2	1.2

#### 3.2.2. Key block analysis.

The 3D visualization program developed in this study was employed to comprehensively search for all independent blocks within the entire spatial domain. The key blocks were determined by considering only the self-weight of the blocks. Initially, deterministic key blocks were identified based on the confirmed large-scale discontinuities, followed by a qualitative prediction of the distribution of random blocks using the simulated discontinuity parameters.

**Fig 14:**
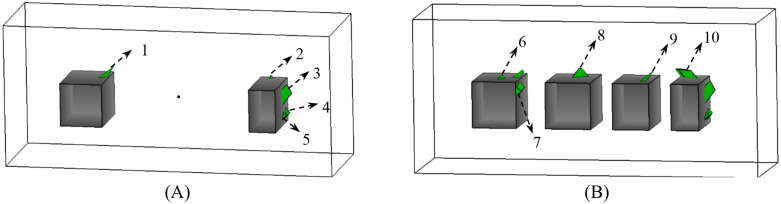
Key blocks generated by deterministic discontinuities after mining phases. **(A)** Distribution after the first mining phase, showing key blocks at stope 1# and 4#. **(B)** Additional key blocks identified after the second mining phase due to excavation-induced discontinuity growth.

Three sets of deterministic discontinuity information were sequentially incorporated into the model. After the first mining phase, software calculations revealed the presence of one key block located atop stope 1# and four key blocks positioned along the sidewalls of stope 4#, as depicted in Fig 14(A). Upon completion of the second mining phase, the deterministic discontinuities underwent interactions with some adjacent random discontinuities, increasing the size of the discontinuities. Following adjustments to the discontinuity dimensions, a renewed search for key blocks was conducted, revealing the addition of two key blocks in stope 1# and one key block in stope 4#, as illustrated in Fig 14(B). The key block information derived from the deterministic discontinuities is summarized in Table 5. These findings align with field observations, indicating that rock mass disturbance due to excavation leads to an increase in discontinuity dimensions and the number of key blocks.

**Table 5:** The key blocks information generated by deterministic discontinuities.

Number		Volume/m^3^	Face quantity	Number of sliding face	Safety factor
**The first mining step**	1	50.03	4	2	0.92
	2	2.68	4	Fall	--
	3	156.97	6	2	0.73
	4	31.25	4	2	0.69
	5	13.89	4	1	0.72
**The second mining step**	6	9.88	4	Fall	--
	7	88.78	6	2	0.91
	8	100.74	5	Fall	--
	9	38.73	4	2	0.96
	10	148.86	6	2	0.74

Due to the large quantity and small size of random discontinuities, accurately measuring their individual properties poses a challenge. Nevertheless, these planes play a significant role in governing the behavior of surrounding rocks. By adopting the quantitative statistical method, they can be described by the disk model and simulated by the Monte Carlo method, and the properties of rock mass structure can be evaluated macroscopically.

**Fig 15:**
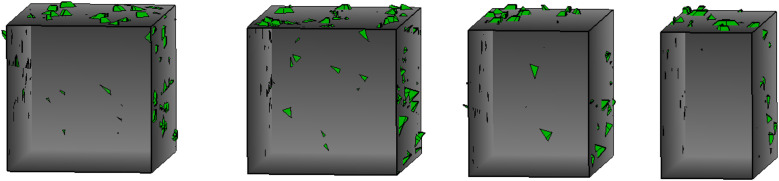
Key blocks formed by random discontinuities, showing distribution along deterministic discontinuities.

Following the identification of key blocks formed solely by the deterministic discontinuities, a comprehensive block analysis was conducted. This analysis was based on an integrated model that combined the deterministic discontinuities (Table 3) with the stochastic discontinuity sets (Table 4). The simulation results, presented in Fig 15, show the 205 key blocks formed by this combined network. For clarity, the key blocks formed only by the deterministic discontinuities have been hidden in this visualization. The analysis indicates that under the influence of random discontinuities, the total number of key blocks is 205, with 124 of them formed by the involvement of deterministic discontinuities, accounting for 60% of the total. The maximum volume of the key blocks is 5.37m³, with an average volume of 2.65m³, and they are primarily distributed on both sides of the deterministic discontinuities. Notably, the northern section of the -40m horizontal middle level exhibits a high concentration of key blocks, warranting special attention, particularly along the deterministic discontinuities. Appropriate investigations and support measures should be implemented in these areas.

This methodology enables the prediction of the key block distribution within the surrounding rocks of the mining field, providing crucial technical support for ensuring safe production and implementing effective support measures. For deterministic blocks, the length, position, and support density of anchor rods can be determined based on the calculated block shape, size, and mechanical parameters of each sliding face. Conversely, for random blocks, a reliability-based approach in support design is employed by analyzing statistical patterns of exposed face area, buried depth, and volume.

## 4. Discussion

### 4.1. Comparative analysis with established block theory approaches

The proposed method addresses a specific gap in traditional block theory, which is most robust when applied to convex or simply-shaped excavations. Conventional algorithms for cutting polyhedra with planes are notoriously complex and less reliable when applied directly to concave bodies. Our strategy of decomposing the problem into tractable convex sub-tasks, followed by a rigorous merging operation, offers a computationally efficient and robust alternative. This “divide-and-conquer” logic, facilitated by auxiliary faces that are later removed, is a key differentiator.

When contrasted with more advanced numerical techniques that model block deformation and complex interactions, such as those using deformable models or incorporating sophisticated inter-block forces and rotation the present method serves a distinct and critical purpose. It excels in the geometric identification phase, providing a complete and accurate inventory of all kinematically admissible blocks. This inventory is a fundamental prerequisite for any subsequent, more computationally intensive mechanical analysis. The integration of this geometric engine into the visual platform GeoSMA-3D, as demonstrated by the clear presentation of block configurations in Figs 13 and 14, significantly enhances its utility for practical engineering assessment and decision-making.

### 4.2. Practical implications for rock engineering support design

The findings from the case study translate directly into actionable engineering insights. The clear distinction between large, deterministic key blocks and smaller, stochastically generated blocks enables an optimized, risk-based support design strategy. For the large, structurally-controlled blocks (e.g., volumes > 5 m³), targeted, high-capacity reinforcement such as long, pre-stressed anchors are justified. In contrast, areas dense with smaller random blocks are better served by systematic support patterns (e.g., shorter bolts with wire mesh). This approach promotes both safety and economic efficiency by allocating resources where they are most effective. The ability to forecast instability zones, as demonstrated in the northern section of the -40m level, allows for proactive support installation, thereby enhancing overall mining safety.

### 4.3. Limitations and avenues for future research

Despite its demonstrated effectiveness in geometric identification and stability assessment under gravitational loading, this study has inherent limitations that outline a clear path for future work. Firstly, the stability analysis is currently based on a rigid-block assumption with only gravitational forces considered. The influence of in-situ stress redistribution due to excavation and potential dynamic loads are not accounted for. Integrating a stress analysis module to determine forces acting on blocks would significantly enhance the mechanical realism of the stability calculations, particularly for deep mining scenarios.

Secondly, the rigid-block model cannot capture progressive failure mechanisms involving block deformation, tensile cracking, or shearing through intact rock. A powerful future extension would be to use the topology of the identified block system as a direct input for a Distinct Element Method (DEM) or Discontinuous Deformation Analysis (DDA) simulation, enabling a fully coupled geometric-mechanical analysis.

Finally, the inherent uncertainty in the parameters of the random discontinuity network, despite being based on field statistics, suggests a natural progression towards probabilistic analysis. Coupling the proposed identification method with a reliability-based approach (e.g., combining with Monte Carlo Simulation) would allow for the calculation of block failure probabilities, thereby providing a more robust framework for risk-informed design decisions.

## 5. Conclusions

This study examines the -40m horizontal middle section of a metal mine in Hebei Province, focusing on the identification and stability analysis of surrounding rock key blocks in the northern mining area. Utilizing a combined approach of field measurements and numerical simulations, the following conclusions are drawn:

(1) By decomposing the complex concave model into several convex bodies, this study avoids the challenges associated with using planes to cut concave bodies in traditional methods. This approach enables precise identification of blocks between complex excavation surfaces and arbitrary structural surfaces.
(2) During mining, the surrounding rocks of adjacent stopes undergo disturbance, leading to an increase in discontinuity dimensions and, consequently, an augmentation in the number of key blocks. Therefore, it is crucial to continuously monitor the development of discontinuities, timely identify blocks, and accurately evaluate the state of surrounding rocks.
(3) In the selected project, there are 124 blocks formed with the participation of deterministic discontinuities, accounting for 60% of the total key blocks. The maximum volume of the key blocks is 5.37m^3,^ with an average volume of 2.65m^3^. The random blocks are mainly distributed on deterministic discontinuities, which require focused exploration and support measures.
